# Gaseous Microemboli in the Cardiopulmonary Bypass Circuit: Presentation of a Systematic Data Collection Protocol Applied at Istituto Cardiocentro Ticino

**DOI:** 10.7759/cureus.22310

**Published:** 2022-02-16

**Authors:** Mira Puthettu, Stijn Vandenberghe, Pietro Bagnato, Michele Gallo, Stefanos Demertzis

**Affiliations:** 1 Cardiac Surgery, Istituto Cardiocentro Ticino, Ente Ospedaliero Cantonale, Lugano, CHE; 2 Biomedical Sciences, Università della Svizzera Italiana, Lugano, CHE; 3 Cardiac Surgery, University of Padua, Padua, ITA

**Keywords:** bubble counter, systematic data collection, cardiopulmonary bypass circuit, cardiac surgery, air emboli

## Abstract

Air emboli are reported to enter the cardiovascular system during cardiac surgery despite air-bubble filters in the arterial line of the cardiopulmonary bypass (CPB). A potential association with stroke, covert cerebral insults and cognitive decline after cardiac surgery has been hypothesized. Although most of the previous studies failed to prove it, this hypothesis cannot be rejected because the situation in the operating room (OR) is multifactorial and complex. Therefore, rigorous and standardized protocols are needed to investigate sources, patterns, as well as effective quantity and volume of air embolism.

We hereby present our protocol in detail for systematic data collection as a standard quality control measure at our center, where air bubbles in the cardiopulmonary bypass circuit are measured by a commercial bubble counter. We also show a preview of the type of information that can be obtained for future analysis. The eventual aim is to determine a potential association between air emboli and adverse postoperative outcomes, as well as to identify major sources of air bubbles generation and in the long run to find effective prevention strategies.

## Introduction

Air micro-embolism reportedly occurs despite up-to-date air filtering in the arterial line of the cardiopulmonary bypass (CPB) circuit [1-2]. The almost spontaneous and logical association with neurological complications occurring after open-heart surgery, which range from 1 to 6% for stroke [3-4] but up to 76% for silent brain micro-infarcts or micro-bleeds [5-6], could not be established beyond doubt. The same applies to the relationship between air micro-embolism and the described cognitive decline affecting a variable percentage of patients after open-heart surgery [7-10]. The pathogenesis of adverse neurologic outcome depends on both patient-related risk factors and procedure-related events, so gaseous microemboli may be only one of the contributing factors to a multifactorial problem (Unpublished original research article: Kopjar T, "Silent Brain Injury Associated to Postoperative Cognitive Decline after Coronary Bypass Surgery", 32nd EACTS annual meeting, 2018). Additionally, the situation in the operating room (OR) involves the patient, the surgical staff, CPB components, the type of surgery and related manual manipulations, which all affect the embolic load on the patient. Microemboli have been proven to be dangerous in experimental and clinical situations without any other confounding factors [11], so awareness and prevention will certainly benefit patients.

The goal of this project is to standardize a method for the investigation of air emboli measured in the CPB circuit during cardiac surgery at our institution. Air bubble number and volume are recorded by a three-channel ultrasonic bubble counter (BCC300, Gampt GmbH, Meerseburg, Germany) and stored together with information on patient, procedure and CPB specifications. This methodology enables us to search for potential correlations between air bubbles and postoperative neurological complications. Moreover, it will allow us to define high-risk factors or maneuvers that can be targeted for future prevention strategies.

## Materials and methods

Data collection

Patient Population

All patients undergoing elective on-pump cardiac surgery at our center were considered for the recording of air bubbles in the CPB circuit. There was no selection based on the type of procedure or any other criteria. All operated patients have given written informed consent regarding the use of their data for clinical research and quality control studies. The study was conducted in accordance with the Declaration of Helsinki, the Cantonal Ethics Committee approved the retrospective use and evaluation of patient-related data (approval number: CE TI 4029).

CPB Circuit and Management

CPB circuit was built with prefabricated standardized kits from four different brands consisting of tubing, heat-exchanger for cardioplegia, venous reservoir, oxygenator and arterial filter. The same circuit scheme was used for all patients.

Priming was done with a solution of Ringer's acetate, mannitol 20% (left out if the patient suffered from renal insufficiency) and heparin (standard value: 10000 IU). Priming volumes ranged between 1200-1300 ml.

All perfusionists used the same standardized de-airing procedure, but de-airing of the arterial filter was performed following the manufacturer’s instructions.

Blood draws and drug administrations were done as usual from a line downstream of the oxygenator (unidirectional flow) and upstream of the venous line.

Generally, when on bypass, the target mean arterial pressure was between 50 and 80 mmHg. Cardiac arrest was induced in all cases with the antegrade infusion of cardioplegic solution (1000 ml, Del Nido’s cardioplegia, 4:1 mixed with arterial blood from the CPB circuit).

Data Acquisition

Bubbles in the CPB circuit were measured by the ultrasonic bubble counter (BCC300, Gampt) throughout the bypass time in three locations via 3/8” tubing clamp-on probes. The BCC300 counted the number of bubbles passing through a probe every second, measured the bubble diameter and derived the bubble volume. The measurable diameter range was 10 to 2000 µm, split in bins of 10 µm, while overrange bubbles (>2000 µm) were also counted but without detailed data. The lower bound of the measuring range was set to 20 µm to exclude noise bubbles. Data were presented as a bubble count histogram on the screen of the device and stored internally. Once terminated, the recording could be exported either as a proprietary format (.gdf) with all the data or as a summary MS Excel file.

The probes were consistently placed according to our protocol as follows: upstream of the oxygenator (blue probe), upstream of the arterial filter (green probe), and upstream of the arterial cannula (red probe), thus measuring the bubbles going directly into the patient. Data recording was usually started immediately (<1 min) before pump start and stopped immediately after pump stoppage. Only the first instance of extracorporeal support was recorded on the BCC300 and repeat on-pump phases were omitted. 

Data Storage

Data were exported once per month from the BCC300 (.gdf) and from the CPB machine (.pdf) and combined with a scan of the cannulation specific data (.pdf) and an electronic logbook with comments from both perfusionists and researchers (.xlsx). At this stage, each such data batch was monitored and checked for signal quality, completeness of records and accordance with protocol. If correctness was confirmed, then data were processed as described below and appended to a master file containing all the recordings acquired to date.

Data processing

For each case, a total of 81 parameters were extracted. The main parameters that are interesting for analysis are shown in Table 1. Other secondary parameters were stored as well to simplify information retrieval, troubleshooting, and assessing inclusion criteria. An anonymized patient ID number and the date of surgery were used for matching the records obtained from the different data sources.

**Table 1:** Main parameters extracted from four data sources. Legend: BCC300: model of bubble counter used, CPB = cardiopulmonary bypass, # = number, BSA = Body Surface Area, BMI = Body Mass Index, CO2 = carbon dioxide.

From BCC300	From CPB machine	From cannulation specifications	From logbook
patient ID, date of surgery	patient ID, date of surgery	patient ID, date of surgery	patient ID, date of surgery
For each patient and each probe:	For each patient:	For each patient:	For each patient:
Count: total # of bubbles, # of overrange bubbles; Volume: total volume of bubbles; Diameters: average (calculated), median (calculated), maximum (measured)	Patient info: height, age, date of birth, weight, gender, BSA, BMI, risk factors; Surgery info: type of surgery, surgical access, target flow of CPB, perfusionist name, surgeon name, CPB duration (+start and stop times), aortic clamp duration (+start and stop times), brand of CPB components, vacuum assisted drainage (yes/no), CO_2_ insufflation (yes/no), total volume of cardioplegia, triggered level alarm in venous reservoir (yes/no), initial hematocrit, number of blood draws, number of drug administrations	Arterial cannula: model, size, cannulation site; Venous cannula: model, size, cannulation site; Patient info: ejection fraction	Comments: from perfusionists, from researchers

BCC300 Data

Each .gdf file was first converted directly in the offline BCC300 software to an MS Excel file containing a worksheet with the histogram of the whole course of recording and a sheet with a time-stamped overview of bubble counts and volumes measured every second. A custom Matlab (The MathWorks Inc., Natick, MA) script was used to extract or derive 20 main parameters (Table 1, column “From BCC300”) from this .xlsx file and to add them to the final master file.

Count histograms were not stored in the database but raw .gdf files were, such that histograms can be reconstructed for some case-specific analysis.

Patient and Surgery Information

Each .pdf file extracted from the CPB machine was processed by a separate Matlab script, which extracts relevant information by text (string) comparison. In this case, 29 main parameters were extracted for the master file (Table 1, column “From CPB machine”).

Cannulation Specifications

Specifications on cannulation were transcribed manually in the master file. Nine main parameters were extracted from the scan (Table 1, column “From cannulation specifications”).

Comments on Data Collection and Data Processing

Comments from perfusionists made during the procedures and from researchers made during the data quality check were directly copied from the logbook into the master file via a Matlab script that matches patient IDs and dates of surgery (Table 1, column “From logbook”).

Preliminary observations

A group of 10 patients who underwent Coronary Artery Bypass Graft (CABG) surgery was selected for this manuscript as an illustration of the type of data that can be obtained with our protocol. For all of them, the arterial cannula was located in the ascending aorta, while the venous cannula was inserted in the right atrium. The subset was obtained from our current database (on the 1st of December 2021: total of 123 records, including both CABG and open-heart-chamber surgery patients). Selection criteria included good quality of signals in the BCC300 recording (no interruptions, absence of interference with other devices and of noise), consistency with the protocol and completeness of records (no missing data from other data sources).

We remind the reader that the purpose of this article is to uniquely present the data collection protocol applied at our center. The analysis on the potential correlation between gaseous emboli and other parameters (for example, perfusionist maneuvers, priming volume and solution, cardioplegia, bypass time, mean arterial pressures or postoperative outcomes) is beyond the scope of this work.

In the next section, we present some graphs obtained from the subset of selected patients to show the type of information that can be obtained for future studies.

## Results

An overview of the patient cohort is shown in Table 2. All patients survived the operation, no stroke or other clinically evident embolic complication was identified. Total bubble counts and volumes measured on the red probe (air bubbles going into the patient) are reported as well. The amount of air bubbles differs largely between cases, both in terms of counts and volumes, even for patients who underwent the same number of bypass grafts. Additionally, it can be noted that bubble count and bubble volume are not linearly proportional: a bigger number of air bubbles does not always translate to higher air volume.

**Table 2:** Information on the 10 selected CABG patients with corresponding total bubble counts and volumes (nL) measured by the red probe. Measuring range of the bubble counter was set to 20-2000 µm. Legend: # = number, EF = Ejection Fraction, CABG = Coronary Artery Bypass Graft.

Patient #	Age	Gender	EF [%]	Type of surgery	Bypass duration [min]	Tot bubble count	Tot bubble volume [nL]
1	76	M	40	CABG x 3	80	69	54
2	62	M	60	CABG x 3	68	1243	281
3	64	M	23	CABG x 4	91	450	5
4	74	M	50	CABG x 4	108	361	1590
5	60	M	60	CABG x 4	94	14	0
6	67	F	60	CABG x 5	127	1104	543
7	59	M	58	CABG x 5	176	3424	278
8	55	M	37	CABG x 5	135	1686	2773
9	71	F	65	CABG x 5	160	3110	313
10	75	M	56	CABG x 5	128	3193	1214

**Figure 1:**
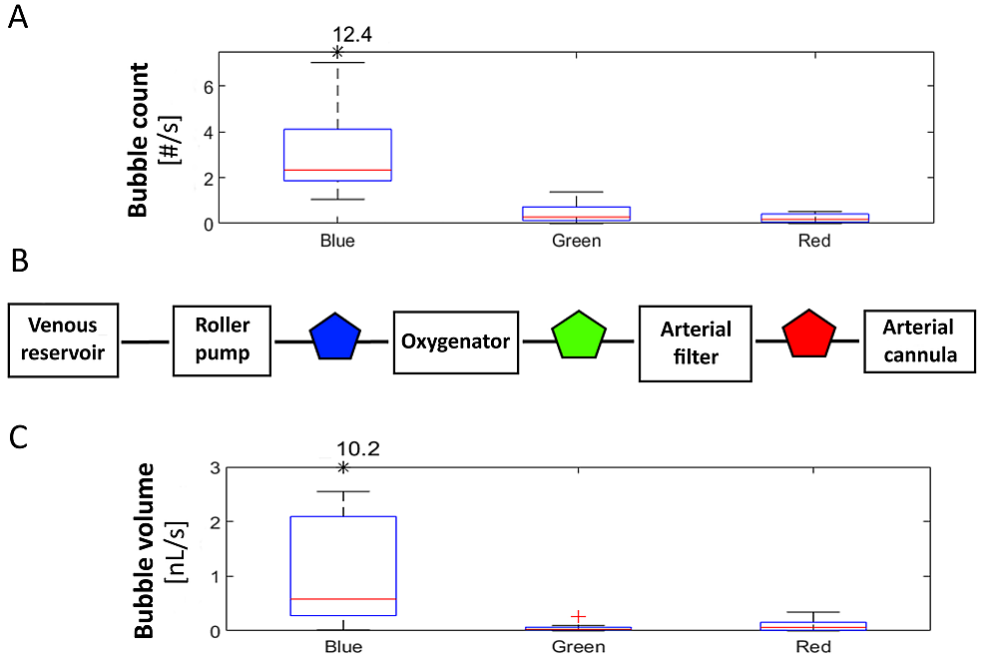
Overview of captured air bubbles in the CPB circuit. A) Boxplot of normalized total bubble counts (average bubble count per second) for all probes. B) Scheme with positions of probes (pentagons) in the CPB circuit. C) Boxplot of normalized total bubble volumes (average bubble volume per second) for all probes. Outliers outside the plot limits are shown as black star (*). Legend: CPB = cardiopulmonary bypass, # = number.

A better overview of captured air bubbles in the CPB circuit is depicted in Figure 1, which shows total bubble counts and total bubble volumes measured at all three recording locations and normalized by the duration of the CPB (average bubble count/volume per second). Some preliminary observations can already be done. A big part of bubbles (both in terms of count and volume) is filtered by the oxygenator, since there is a clear drop in measurements obtained with the blue probe versus measurements obtained with the green probe. The same pattern is not found after the arterial filter (green vs. red probe), where only a reduction in the number of bubbles is observed, but the volume, in contrast, increased. Since the main purpose of this manuscript is to show the data collection principle and to illustrate the type of data that can be obtained, no statistical tests were performed on this data subset.

**Figure 2:**
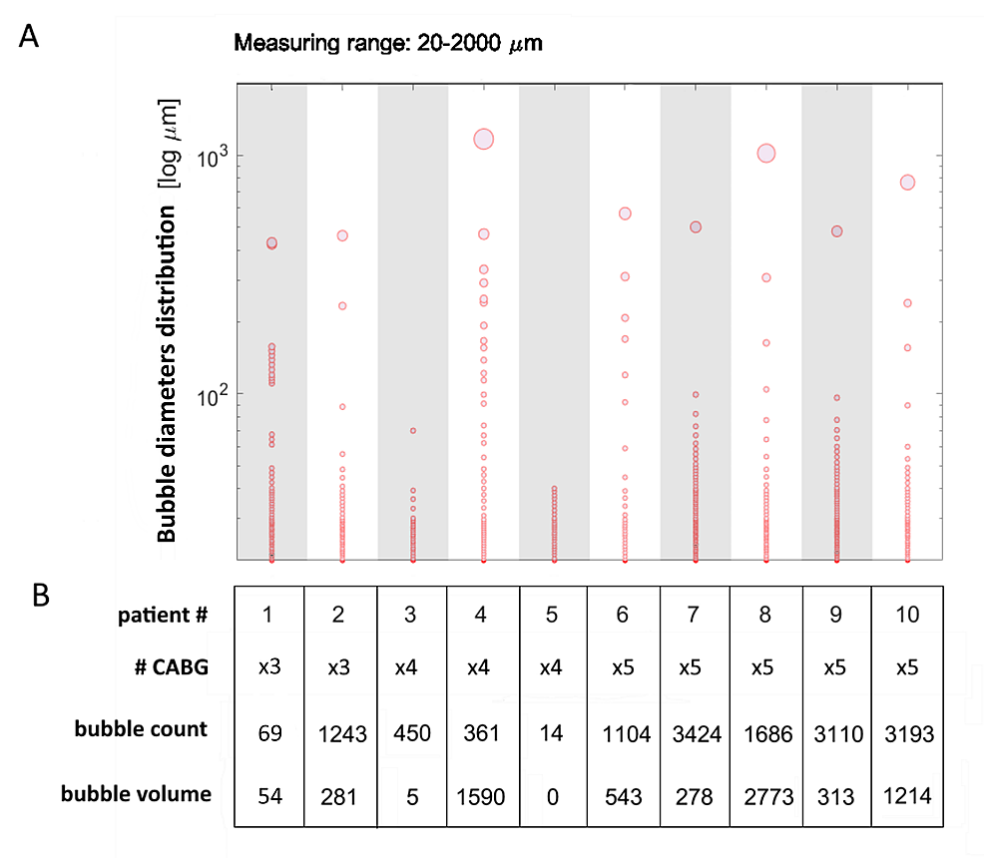
Distribution of bubble sizes. A) Distribution of bubble diameters (log scale) for each patient expressed in percentiles (100 data points/patient). The highest data point indicates the maximum bubble diameter (100th percentile) measured in that patient. B) Patient information, including total bubble count and total volume (nL) measured on the final (red) probe upstream of the arterial cannula. Legend: # = number, # CABG = number of Coronary Artery Bypass Grafts.

Another aspect that can be investigated is the distribution of bubble sizes. Figure 2A presents a plot with percentiles of bubble diameters for each selected patient and for the red probe only. Thus, with 100 calculated data points per patient (instead of the thousands that were actually measured) where the highest data point effectively represents the maximum measured bubble diameter (100th percentile: 100% of the bubbles were smaller or equal to the percentile) that flowed through the arterial cannula. It can be observed that the distribution is also different for each patient, even if they underwent the same type of surgery in this subset.

Figure 2B reports the number of bypass grafts the patient received, bubble count and bubble volume. The distribution of bubbles for patients who received the least air volumes (patient #3 and patient #5) is skewed towards the lower range (mainly below 40 µm). Two out of ten patients received one bubble with a diameter bigger than 1 mm (100th percentile above 1 mm), which results in the two highest air volumes (patient #4 and patient #8). On the other hand, patients with comparable total bubble counts can have completely different bubble diameter distributions. For example, patient #3 and patient #4 received 450 and 361 bubbles, respectively, but much bigger bubbles are observed in patient #4.

**Figure 3:**
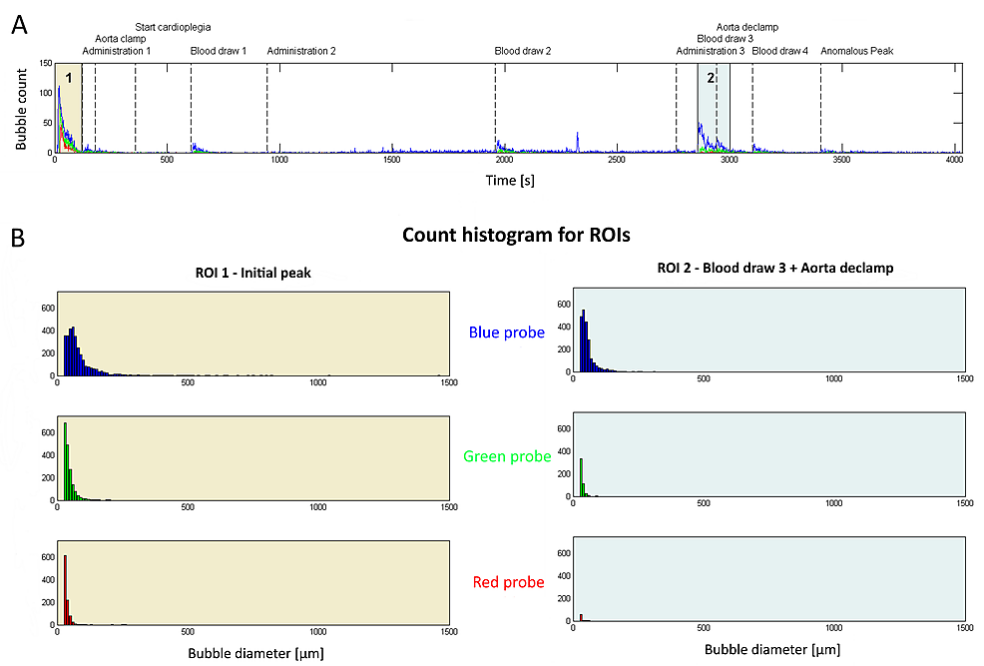
Example of BCC300 recording with bubble count over time for patient undergoing CABGx3 (patient #2). A) Bubble count over time for all probes during the whole on-pump period is shown. Events during surgery are indicated as vertical dashed lines. Two regions of interest (ROIs) are highlighted as shaded area. B) The previous highlighted ROIs are detailed by their histograms, where ROI 1 represents the first two minutes from CPB start. Legend: ROI = Region Of Interest, CABG: Coronary Artery Bypass Graft, BCC300: three-channel ultrasonic bubble counter (BCC300, Gampt GmbH, Meerseburg, Germany).

Preliminary observations can also be performed for case-specific investigation. Figure 3 shows what happened during the surgery of patient #2 (CABG x 3, the shortest CPB period in the selected subset), where several peaks can be detected in the count-rate timeline. The main events reported in this timeline are extracted from the CPB machine record. It should be noted that routine and supposedly harmless perfusionist maneuvers such as drug administration or blood draw from the venous reservoir in fact generate a cloud of bubbles that travels towards the oxygenator (blue probe). Count histograms for selected regions of interest (ROIs, Figure 3B) show that the distribution of bubbles within each ‘cloud’ is event dependent. A wider spectrum is observed during the initial peak (ROI 1) where bubbles from 50 to 60 µm in diameters were most frequent, while during the multi-factorial event of ROI 2 (Blood draw 3 + Aorta de-clamp), the distribution is shifted to the left where 30-40 µm was the most populated diameter bin.

**Figure 4:**
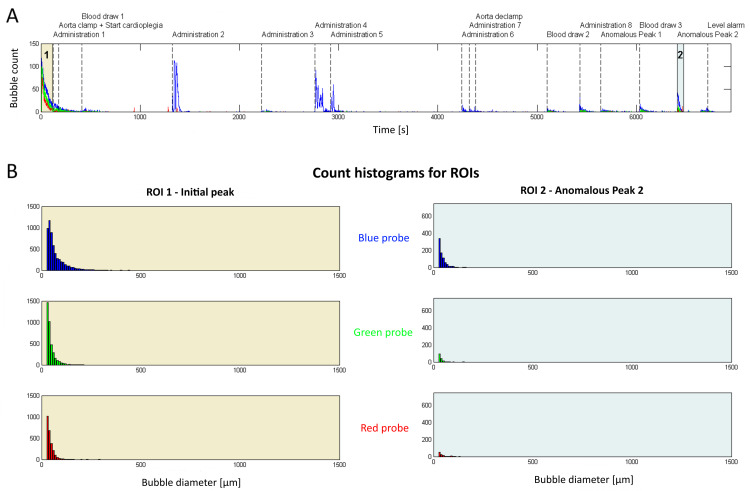
Example of BCC300 recording with bubble count over time for patient undergoing CABGx5 (patient #7). A) Bubble count over time for all probes during the whole on-pump period is shown. Events during surgery are indicated as vertical dashed lines. Two regions of interest (ROIs) are highlighted as shaded area. B) The previous highlighted ROIs are detailed by their histograms, where ROI 1 represents the first two minutes from CPB start. Legend: ROI = Region Of Interest, CABG: Coronary Artery Bypass Graft, BCC300: three-channel ultrasonic bubble counter (BCC300, Gampt GmbH, Meerseburg, Germany).

Another example is shown by Figure 4, where patient #7 underwent CABG x 5. In this case, the highest total number of bubbles was observed through the arterial cannula (3424 versus 1243 in patient #2). Nevertheless, total air volume was comparable with patient #2 (281 nL versus 278 nL, respectively). Some variability is observed between peaks. The initial peak (ROI 1, first two minutes of CPB) is different from the previous patient; it is drawn out longer and there is almost double the number of bubbles (note the different scale in the figure) and the count histogram is shifted to the left, indicating mostly small bubbles. The second ROI shows the number of bubbles for an anomalous peak, meaning that the cause of it cannot be correlated to a specific event. Count histogram is shifted even more to smaller bubbles with a peak at 20-30 µm diameters, which is in the magnitude of baseline noise. However, the maximum bubble diameter that travelled through the arterial cannula for this patient was 500 µm, while for the previous patient it was only 460 µm.

From assessing the previous two figures, it is shown that downstream travel through the CPB system from blue to green to red probe results in bubble reduction in numbers. Still, by comparing the left side of Figures 3B, 4B, it can be observed from these two examples that a non-negligible number of bubbles is generated during the first two minutes of CPB and a relevant portion of it persists at the level of the red probe, while other events appear to push fewer, smaller bubbles into the patient.

It should also be noted that the patients represented in Figures 3-4 are not at all extreme cases since we sometimes observe surgeries where unexpected bubbles >1 mm in diameter travel towards the arterial cannula, which was not the case in these two examples. Patient #8 is one of the cases with at least one bubble >1 mm in diameter and the one case with the highest recorded air volume. Besides the high number of perfusionist maneuvers (12) reported in this surgery, the mean flow rate was also lower than usual (requested by the surgeon at four different moments). While for patient #4 (the other patient with at least one bubble >1 mm), the level alarm in the venous reservoir was triggered two times during the bypass period, but this also happened in cases with smaller total bubble volumes.

## Discussion

Air emboli generated during cardiac surgery are one of the potential causes for neurological adverse outcomes in the post-operative period, such as cognitive decline and stroke, and are also responsible for cardiac events during surgery [12]. Up until now, two major sources of air emboli have been identified: manipulations of the opened heart and arteries via direct blood-air contact, and manipulations on components of the CBP circuit [13].

Our preliminary observations from consistent measurements performed with the BCC300 ultrasonic bubble counter show that air bubbles are indeed produced in the CBP system during cardiac surgery and a portion proceeds to enter the patient’s arterial system. Moreover, surgical manipulations on the heart and arteries even with the heart closed result in clouds of air emboli traveling through the system and (re)-entering the patient.

Peaks in the count-rate of air bubbles are mostly visible following manipulations on the CPB system itself such as administrations of drugs and blood draws from the venous reservoir, but also following surgical manipulations which lead to a rapid decrease in the blood level in the venous reservoir triggering the alarm. Large bubble clouds were also observed at the beginning of the bypass period, despite all the effort of the perfusionist to de-air the circuit. However, it is important to note that not all bubbles entering the body are dangerous: small bubbles <40 µm in diameter dissolve in blood in less than 30 seconds and can therefore do little damage to well-perfused organs [1]. Nevertheless, a scientifically based threshold to distinguish between air bubbles (or bubble clouds) that are dangerous and those that are harmless is still to be defined, whereby bubble clouds should be characterized in terms of counts, volumes, and diameters.

Count-rate peaks observed with the BCC300 are always different, showing variance between patients but also within a single patient. This confirms that the situation in the OR is multifactorial and complex. Variance at the input, as well as variance at the output, makes it difficult to investigate the issue of air emboli production during cardiac surgery, let alone describe methods for improvement. However, some previous studies attempted to do so, resulting in interesting findings.

In the study of Chung et al. [1], air volumes were measured in the middle cerebral artery territories using transcranial Doppler. They found that several surgical manipulations, both in CABG and open-heart-chamber surgeries, generated air bubbles in the brain. In their CABG patients, the biggest bubbles were produced in the period after removal of aortic cross-clamp, especially following deairing and side biting clamping off. Even more air bubbles were found in patients undergoing open-heart surgery. Similar findings were obtained in their following study [8]. Interestingly, bubbles in the brain due to the administration of drugs or blood draws were reported only once. We may assume that these air bubbles, entering the systemic circulation via the arterial cannula, either dissolve before reaching cerebral regions or travel towards other organs, but additional data are necessary to confirm this, especially since both measuring methods have a very different scope.

Related to our observations from CPB component manipulations, Borger et al. showed a difference in neuropsychological outcome when comparing patients who underwent surgery with less than 10 perfusionist interventions versus those with 10 or more [14]. An overview of possible events and variables that can impact the embolic load is presented by Lou et al. [13], where perfusionist and surgical interventions are listed, as well as CPB components and management.

Our preliminary observations are consistent with previous studies. A clear understanding of air emboli generation is needed to apply effective prevention strategies. Absolute values of bubble counts and bubble volumes are good parameters to have an overview of the bubble load during surgery. But it is also possible to determine the bubble diameter distribution and the same analysis can be done for specific regions of interest. This allows both a general analysis on the on-pump period or a specific investigation of single event effect.

Nevertheless, this project was designed to address a multifactorial problem, which brings some limitations. The final embolic load during cardiac surgery is possibly dependent on a big number of factors, so, a large sample size is needed to find a potential correlation between the outcome measure (total number of air emboli) and each of the single factors. However, we think the effort will pay off with time. Another limitation comes from the lack of previous standardized methods for bubble recording in the existing literature (at least to our knowledge), which lead to a certain level of subjectivity (for example, during data monitoring and selection of “good” records). With time and experience, we also hope to standardize this stage. The last limitation is due to the complex data collection and processing procedure, which is done by multiple people at different times (data collection during cardiac surgery vs data processing once per month). Good communication between parties is required to ensure good quality of data to be analyzed in the future, and therefore the logbook was created specifically for this purpose. Another aspect that needs more attention is the necessity to define a threshold to differentiate between harmless air bubbles and dangerous ones. Small air bubbles may dissolve in blood before reaching the brain or other organs and do damage, but more data are required to define the cut-off diameter.

## Conclusions

Consistent measurements on the CPB circuit with an ultrasonic bubble counter and subsequent analysis is a powerful tool that can bring more insight to the multifactorial problem of air emboli generation during cardiac surgery. This standardized method to detect air bubbles during cardiac surgery can be applied in a large population to investigate the correlation between air bubbles with clinical variables. In future studies, we aim to use this tool to explore solutions for reducing the amount of dangerous air emboli and to make cardiac surgery safer for the patient.
